# Unplanned readmission rates, length of hospital stay, mortality, and medical costs of ten common medical conditions: a retrospective analysis of Hong Kong hospital data

**DOI:** 10.1186/1472-6963-11-149

**Published:** 2011-06-17

**Authors:** Eliza LY Wong, Annie WL Cheung, Michael CM Leung, Carrie HK Yam, Frank WK Chan, Fiona YY Wong, Eng-Kiong Yeoh

**Affiliations:** 1 School of Public Health and Primary Care, The Chinese University of Hong Kong, Prince of Wales Hospital, Shatin, N.T., Hong Kong

## Abstract

**Background:**

Studies on readmissions attributed to particular medical conditions, especially heart failure, have generally not addressed the factors associated with readmissions and the implications for health outcomes and costs. This study aimed to investigate the factors associated with 30-day unplanned readmission for 10 common conditions and to determine the cost implications.

**Methods:**

This population-based retrospective cohort study included patients admitted to all public hospitals in Hong Kong in 2007. The sample consisted of 337,694 hospitalizations in Internal Medicine. The disease-specific risk-adjusted odd ratio (OR), length of stay (LOS), mortality and attributable medical costs for the year were examined for unplanned readmissions for 10 medical conditions, namely malignant neoplasms, heart diseases, cerebrovascular diseases, pneumonia, injury and poisoning, nephritis and nephrosis, diabetes mellitus, chronic liver disease and cirrhosis, septicaemia, and aortic aneurysm.

**Results:**

The overall unplanned readmission rate was 16.7%. Chronic liver disease and cirrhosis had the highest OR (1.62, 95% confidence interval (CI) 1.39-1.87). Patients with cerebrovascular disease had the longest LOS, with mean acute and rehabilitation stays of 6.9 and 3.0 days, respectively. Malignant neoplasms had the highest mortality rate (30.8%) followed by aortic aneurysm and pneumonia. The attributed medical cost of readmission was highest for heart disease (US$3 199 418, 95% CI US$2 579 443-803 393).

**Conclusions:**

Our findings showed variations in readmission rates and mortality for different medical conditions which may suggest differences in the quality of care provided for various medical conditions. In-hospital care, comprehensive discharge planning, and post-discharge community support for patients need to be reviewed to improve the quality of care and patient health outcomes.

## Background

Hospital readmission rates have been proposed as an important indicator of patient health outcome and healthcare system performance [1-4], and they are also regarded as an indicator of poor care or a lack of coordination of care services. The costs associated with readmission are also very high [5]. In a report by the World Health Organization in 2005, readmission was cited as a key undesirable outcome of healthcare systems, and reducing readmission rates was cited as one of the top strategic priorities [1], with which Hong Kong concurred. Public healthcare services in Hong Kong are heavily subsidized by the government. Eligible patients pay approximately US$13 per hospital day including all types of services and medication, and all fees and charges are waived for those on public assistance [6]. The increasing readmission rates will have a significant impact on future health-care costs in Hong Kong.

Many different programs for discharge planning and community-based support have been established to reduce hospital readmission rates. However, despite the success of efforts in launching the programs, their effectiveness in reducing readmission rates are inconclusive [2,7-9], and a number of studies have shown no cost savings to service providers [10-15]. Several reports suggested that programs aimed at reducing readmission rates should focus on those conditions at high risk of readmission [12,16-19]. Thus, it would be desirable to identify such conditions to guide efficient resource utilization, and to support the cost of programs and services to those likely to benefit most [20]. Numerous publications have investigated patient factors associated with readmission; the findings show that age, gender, marital status, insurance status, socioeconomic status, employment status, living conditions, and discharge destination are possible predictors of readmission [1-3,21-26]. A number of studies suggested that a focus on patients with defined medical conditions would yield a higher reward in terms of reducing readmissions in the general inpatient population [27]. Although there is literature on readmissions attributed to particular medical conditions, especially heart failure, few of these studies addressed the broader issues involving the impact of variations in disease conditions on readmission, and the implications on health outcomes and costs.

In this study, we examined two key aspects: (1) patient characteristics in 10 common medical conditions and their impact on unplanned hospital readmission; and (2) the implications in terms of health outcomes and costs.

## Methods

### Study Design

The Hong Kong Hospital Authority (HA) computer-based clinical database (Clinical Disease Management, CMS) for calendar year 2007 was used to examine readmission patterns, related disease profiles, health outcomes, and costs in the study. The HA as a public organization is responsible for more than 90% of hospital services in Hong Kong, and hospital services are organized into seven organizational clusters, each serving a geographical region with a population of around one million. Each cluster has one or more acute hospitals that admit seriously ill patients from emergency units, and one or more rehabilitation hospitals which admit transfer patients from the acute hospitals for rehabilitation and convalescence. The CMS computer system includes the patient's demographic data and clinical data upon admission and discharge. The principal diagnosis was coded at discharge according to the World Health Organization 9th International Classification of Disease (ICD-9).

### Study Population

All patients admitted to the wards of the Internal Medicine departments of all public hospitals in Hong Kong in 2007 were identified. Day patients, defined as having a patient-stay of less than 24 hours, were excluded.

### Definition of unplanned readmissions

To determine patients with unplanned readmissions, the first or initial instance in a series of hospitalizations was identified. The first hospitalization in 2007 was identified as the index hospitalization, and a 30-day unplanned readmission was defined as a subsequent or unscheduled admission to the same specialty through the Accident & Emergency Department within 30 days of the index hospitalization [28].

The 30-day timeframe is commonly used in studies in the United States [29-32], whereas a 28-day timeframe is commonly used in the United Kingdom [33-36]. Based on statistical modelling, such as survival analyses as well as sensitivity and specificity analyses, two studies had mathematically demonstrated that 30 days was an optimal choice for identifying readmission rates [37,38]. Thus, we used 30 days as one of the criteria to define unplanned readmission.

### Selection of Medical Conditions

For analysis of the disease in relation to readmission, 10 medical conditions with the highest burden of mortality and morbidity in Hong Kong were chosen for this study: (1) malignant neoplasms (ICD9: 140-208); (2) heart diseases, including hypertensive heart disease (ICD9: 390-429); (3) cerebrovascular disease (ICD9: 430-438); (4) pneumonia, all forms (ICD9:480-486); (5) injury and poisoning (ICD9: 800-999); (6) nephritis, nephritic syndrome and nephrosis (ICD9: 580-589); (7) diabetes mellitus (ICD9: 250); (8) chronic liver disease and cirrhosis (ICD9: 571); (9) septicaemia (ICD9: 038); and (10) aortic aneurysm (ICD9: 441) [39].

### Statistical Analysis

The admission profile of patients, including patients' characteristics and clinical health outcomes of the hospitalization (30-day unplanned readmissions vs. other admissions) were determined and differences were evaluated using the chi-square test or Student *t*-test. For patients with 30-day unplanned readmission, length of hospital stay (LOS) and health outcomes (alive or death) for each medical condition were evaluated. Any admission which was within 30 days of the previous discharge (index admission) was counted as unplanned readmission. A hospital transfer was not considered as a discharge case.

To explore the excess risk of unplanned readmission caused by each medical condition, the disease-specific odds ratios (ORs) for 30-day unplanned readmission were estimated using logistic regression analysis, and all ORs were adjusted for age, gender, receipt of public assistance, residence in an elderly care home, residential district, and cluster of the admitting hospital. Applying attributable risk analysis, we calculated the fraction of unplanned readmissions which could be attributed to each of the selected medical conditions. We then determined the attributable costs using a value of US$486 for an acute bed and US$233 for a long-stay bed, and an exchange rate of US$1 to HK$7.8 [6]. The attributable costs were calculated only for conditions with an OR for hospital readmission significantly greater than 1. To calculate a range for the best estimate of costs, we used the upper and lower limits of the 95% confidence interval (CI) for risk estimates. We used Stata version 10.0 (StataCorp, College Station, Tex) for all statistical analysis.

## Results

### Overall Patient Characteristics and Health Outcomes

There were 337 694 hospital admissions in Internal Medicine in all 38 public hospitals in Hong Kong in 2007. As shown in Table 1, the mean age of the patients was 69.5 years (standard deviation (SD) 17.4), 52.7% were men, 30.8% received public assistance, 19.5% resided in an elderly care home, and the mean mortality rate was 5.1%. The mean LOS was 5.9 days, with the proportion of acute stay patients being 88.1% and rehabilitation stay patients being 11.9%. The admission rates were similar in the seven geographical clusters, ranging from 9.3% to15.3%, except Cluster 2 which had a particularly high admission rate of 29.1%. The overall proportion of 30-day unplanned readmission episodes was 16.7%.

**Table 1:** Characteristics of patients according to types of hospital admission* in Hong Kong in 2007

	Unplanned Readmission n(Col %)	Other Admission n(Col %)	Total n(Col %)	**P-value**^†^
	**(N = 56,329)**	**(N = 281,365)**	**(N = 337,694)**	

**Age **(n)	56,328	281,307	337,635	<0.001
(mean, SD)	75.2(14.6)	68.4(17.7)	69.5 (17.4)	
**Sex**				
Male	30,274(53.7)	147,684(52.5)	177,958(52.7)	<0.001
Female	26,055(46.3)	133,681(47.5)	159,736(47.3)	
**Public assistance**^‡^				
Yes	24,967(44.3)	78,954(28.1)	103,921(30.8)	<0.001
No	31,362(55.7)	202,411(71.9)	233,773(69.2)	
**Lived in elderly home**				
Yes	19,462(34.6)	46,229(16.4)	65,691(19.5)	<0.001
No	36,867(65.5)	235,136(83.6)	272,003(80.6)	
**Admitted cluster**				
Cluster 1	9,018(16.0)	42,536(15.1)	51,554(15.3)	<0.001
Cluster 2	16,467(29.2)	81,699(29.0)	98,166(29.1)	
Cluster 3	7,826(13.9)	35,170(12.5)	42,996(12.7)	
Cluster 4	6,260(11.1)	35,727(12.7)	41,987(12.4)	
Cluster 5	6,290(11.2)	31,615(11.2)	37,905(11.2)	
Cluster 6	4,423(7.9)	26,824(9.5)	31,247(9.3)	
Cluster 7	6,045(10.7)	27,794(9.9)	33,839(10.0)	
**Length of stay**				
Acute bed day (n)	56,324	281,343	337,667	<0.001
(mean, SD)	5.8(7.4)	5.0(7.4)	5.2(7.4)	
Sub-acute bed day (n)	56,329	281,365	337,694	<0.001
(mean, SD)	0.8(9.8)	0.7(9.3)	0.7(5.2)	
Total bed day (n)	56,324	281,343	337,667	<0.001
(mean, SD)	6.6(9.8)	5.8(9.3)	5.9(9.4)	
**Health Outcome**				
Live	51,312(91.1)	269,120(95.7)	320,432(94.9)	<0.001
Dead	5,012(8.9)	12,223(4.3)	17,235(5.1)	

*Hospital admissions were grouped into two types: (1) Unplanned readmission - the patient re-admitted again to hospital within 30 days after his/her last hospital admission & (2) Other admission - included all other hospital admitted episodes except the unplanned readmission one.

†Chi-square tests/T-tests

‡only include those who are receiving assistance from the government such as Comprehensive Social Security Assistance (CSSA) in Hong Kong

### Differences between 30-day Unplanned Readmissions and Other Admissions

Table 1 presents comparisons of patient characteristics and health outcomes between 30-day unplanned readmission episodes and other admission episodes. Patients with unplanned readmission were significantly older (mean age 75 years; SD 14.6 years, *p *< 0.001), and were more likely to be male (53.7%, *p *< 0.001), to receive public assistance (44.3%, *p *< 0.001), and to live in an elderly care home (34.6%, *p *< 0.001). The mortality rate of unplanned readmissions was 8.9% and the mean LOS was 6.6 days (SD 9.8 days). Both the mortality rate and mean LOS for unplanned readmissions were statistically significant higher and longer than all other admissions (*p *< 0.001). The readmission rates were also significantly different (*p *< 0.001) in different clusters, with Cluster 1 (16.0%), 3 (13.9%), and 7 (10.7%) having higher rates than the others.

### 30-day Unplanned Readmission-Related Mortality Rates for each Medical Condition

In the 30-day unplanned readmission group, the mortality rates for the selected medical conditions ranged from 1.8% to 30.8% (Table 2). The medical condition with the highest mortality rate was malignant neoplasms (30.8%), followed by aortic aneurysm, pneumonia, chronic liver disease and cirrhosis, nephritis, nephritic syndrome and nephrosis, septicaemia, cerebrovascular disease, heart disease, injury and poisoning, and diabetes mellitus.

**Table 2:** Health outcome (death %) in 10 medical conditions of hospital unplanned readmission

Principal Diagnosis (ICD9)	Total n(%)
Malignant neoplasms(140-208)	685(30.8)
Heart disease(390-429)	679(8.0)
Cerebrovascular disease(430-438)	192(13.1)
Pneumonia(480-486)	1,601(24.0)
Injury and poisoning(800-999)	48(5.5)
Nephritis nephritic syndrome and nephrosis(580-589)	269(15.1)
Diabetes mellitus(250)	16(1.8)
Chronic liver disease and cirrhosis(571)	37(16.4)
Septicaemia(038)	148(14.2)
Aortic aneurysm(441)	8(24.2)

### Excess Risk of Unplanned Readmission in each Medical Condition and its Costs

Table 3 shows that the adjusted excess risks of 30-day unplanned readmission among the 10 medical conditions. The risk-adjusted OR of unplanned readmission was significantly greater than 1 for five medical conditions: malignant neoplasms, heart disease, pneumonia, chronic liver disease and cirrhosis, and septicaemia. However, the ORs of unplanned readmission for cerebrovascular disease, aortic aneurysm, diabetes mellitus, and injury and poisoning were significantly less than 1.

**Table 3:** Disease-specific* odd ratios^† ^of hospital unplanned readmission

Principal Diagnosis	OR(95% CI)
Malignant neoplasms	1.22(1.17-1.28)**
Heart disease	1.14(1.11-1.17)**
Cerebrovascular disease	0.45(0.42-0.47)**
Pneumonia	1.13(1.09-1.16)**
Injury and poisoning	0.85(0.79-0.91)**
Nephritis nephritic syndrome and nephrosis	1.01(0.96-1.07)
Diabetes mellitus	0.73(0.68-0.78)**
Chronic liver disease and cirrhosis	1.61(1.39-1.87)**
Septicaemia	1.19(1.11-1.30)**
Aortic aneurysm	0.59(0.41-0.84)**

*The classifications were based on the principal diagnosis of the admission and the specific disease was compare to other diseases of the admission.

†All odd ratios were adjusted for age, gender, received any public assistant, whether lived in elderly home, living district and cluster of admitted hospital in Hong Kong.

**Significant at P < 0.05

As shown in Table 4, the mean LOS for the 10 medical conditions varied from 5.6 to 9.9 days. Patients with cerebrovascular disease had the longest mean LOS, with mean acute and rehabilitation stays of 6.9 and 3.0 days, respectively. Patients with diabetes mellitus had the shortest hospital stay, with mean acute and rehabilitation stays of 4.9 and 0.7 days, respectively. From the results in Table 3, we determined the attributable cost of unplanned readmission for the five medical conditions with high ORs for hospital readmission. The attributable cost of 30-day unplanned readmission was highest for heart disease (US$3,199,418), followed by pneumonia, malignant neoplasms, septicaemia, and chronic liver disease and cirrhosis (Table 4).

**Table 4:** Hospital associated burden due to hospital unplanned readmission among 10 medical conditions*

Principal Diagnosis	Mean(SD) los of total bed	Mean(SD) los of acute bed	Mean(SD) los of sub-acute bed	US$(95% CI)
Malignant neoplasms	9.8(12.9)	8.5(11.0)	1.4(5.8)	1,683,692 (1,317,710-2,032,617)
Heart disease	6.2(7.5)	5.6(5.8)	0.6(4.2)	3,199,418 (2,579,443-3,803,393)
Cerebrovascular disease	9.9(13.2)	6.9(7.9)	3.0(9.7)	---
Pneumonia	7.6(10.4)	6.4(7.5)	1.2(6.8)	2,353,652 (1,768,964-2,920,557)
Injury and poisoning	7.4(10.7)	6.9(10.0)	0.5(3.7)	---
Nephritis nephritic syndrome and nephrosis	7.6(10.2)	7.1(9.2)	0.5(3.9)	---
Diabetes mellitus	5.6(6.4)	4.9(4.3)	0.7(4.4)	---
Chronic liver disease and cirrhosis	7.3(8.4)	7.0(7.5)	0.4(2.5)	292,627 (215,732-358,836)
Septicaemia	8.1(10.2)	7.3(8.5)	0.8(5.0)	605,398 (365,516-828,470)
Aortic aneurysm	8.2(8.9)	7.7(8.8)	0.5(1.9)	---

*The attributable costs were calculated only for the diseases with the odd ratios of hospital readmission were greater than 1

## Discussion

### Principal Findings

In this cohort study of 337 694 hospital admissions in Internal Medicine in Hong Kong, a moderate 30-day unplanned readmission rate was identified. Our findings showed associations between patient demographics and geographical hospital cluster and 30-day unplanned readmission. After adjusting for these risk factors, patients with chronic liver disease and cirrhosis were more likely to have unplanned readmission. Patients with cerebrovascular disease were least likely to have unplanned readmission but had the longest LOS of 10 days. Malignant neoplasms had the highest mortality rate followed by aortic aneurysms and pneumonia. The attributed medical cost of 30-day unplanned readmission was highest for heart disease.

### Comparison with Other Studies

A moderate 30-day unplanned readmission rate of 16.7% was identified, compared with an overall 30-day readmission rate of 5-29% in the United States [40], an overall 28-day readmission rate of 15.3% in the United Kingdom [41], and a 42-day readmission rate of 39-59% for patients discharged from a department of internal medicine in Switzerland [42]. The differences probably arise from variations in methods and definitions, study criteria, and population groups with unplanned readmission. The differences may also be related to the quality and organization of health care, and the availability of social support services. In line with other studies, risk factors associated with 30-day unplanned readmission were male sex, older age, and lower socioeconomic status. Patients who were discharged to elderly care homes had a higher readmission rate than those discharged to their own homes. This could be related to this population being older and having a poorer health status, with a need for more psychosocial support, but could also be a reflection of the quality of care in elderly homes. This problem of unplanned readmission from elderly homes has been referred to as the "revolving door syndrome" in one review [43]. An in-depth study on the quality of care in elderly homes is needed to address this issue. Our study also showed variations in the unplanned readmission rates by geographical hospital cluster, with a higher rate of readmission in Clusters 1, 3 and 7, in which the patients were older and poorer, and in need of residential care, thereby confirming the value of demographic factors in predicting the risk of readmission.

Our study showed that there was a significant difference in LOS and mortality rates between 30-day unplanned readmissions and all other admissions. The longer LOS and higher mortality rate suggests that patients readmitted within 30-days of discharge had a poorer functional status. After malignant neoplasms, the top three high-mortality medical conditions were aortic aneurysms, pneumonia, and chronic liver disease and cirrhosis. The results are in line with other studies suggesting that these conditions were associated with higher mortality as a result of unmet clinical needs and clinical complications (e.g., the need for surgery, infection, postoperative complications) [44-47]. However, Silber et al found that the mortality rate may also be correlated with the failure rate (the tendency for patients with complications to die) as well as unmet clinical needs and complication rate [48,49]. Thus, it seems that the ability to discharge surviving patients who have experienced complications represent two different complementary components of quality that should both be identified when assessing quality of care. Comparing the 10 medical conditions, patients with cerebrovascular disease had the longest LOS, especially in rehabilitation institutions, probably because they required more residential medical rehabilitation and psychosocial support before discharge. While this medical condition had the longest hospital stay, the OR of hospital readmission was, however, less than 1. To maximize the quality of care, future studies need to determine why patients with cerebrovascular disease required a longer hospital stay but had lower unplanned readmission rates, and whether a longer hospital stay may facilitate better discharge planning, community support for psychosocial and rehabilitation care, and subsequently lower readmission rates. Echoing this thought, while patients with heart diseases had the shortest hospital stay in both acute and rehabilitation care, the OR of hospital readmission for heart diseases was greater than 1. Thus, future studies may need to review whether early discharge contributed to hospital readmission in some conditions.

After controlling for significant patient risk factors and geographical cluster, chronic liver disease and cirrhosis, malignant neoplasms, septicaemia, and heart diseases had high attributable risk for 30-day unplanned readmission. Stroke, diabetes mellitus, injury and poisoning, and aortic aneurysm had a lower attributable risk for 30-day unplanned readmission. In the past 10 years, there have been many hospital-based and community support programs introduced to help patients with stroke and diabetes mellitus, including specific "stroke wards" to enhance the rehabilitation of patients, and diabetes mellitus clinics to provide more frequent post-discharge support for patients. The outcomes indicate that a range of disease-specific programs can affect the rate at which patients are admitted for a second time. It is surprising that heart disease still ranks high in the 30-day unplanned readmission rates, even though there are many in-hospital and community programs established for such patients. The outcomes may be related to the life-threatening nature of heart disease. Further study is needed to explore the relationship between readmission rates and heart disease because the disease code includes a number of distinct forms of heart disease. For efficient use of resources in rehabilitation, it would be desirable to identify the patients at risk of readmission, and focused care, including comprehensive discharge planning, community support, and medical follow-up, should be reviewed for the five medical conditions with high risk-adjusted ORs for 30-day unplanned readmission.

Finally, we found that the attributed cost of 30-day unplanned readmission was high. The HA spent approximately US$8 million on unplanned readmissions within 30 days of discharge for five medical conditions in Hong Kong 2007. With a conservative approach, an unchanged readmission rate and inflation of 4.5%, it is projected that the HA will spend US$13 million on 30-day readmissions in 2017, with the cost being much higher when considering readmissions that occurred within 60 and 90 days. Over 10 years, the projected cost increase is 63%, mainly related to the growth of the Hong Kong population, which is projected to increase from 6.9 million in 2007 to 7.5 million in 2017 [50], and HA expenditure on readmissions in future could well exceed our current estimates. Across the 10 common medical conditions, heart disease, pneumonia and malignant neoplasms contributed the greatest financial pressure to the healthcare system. The patient population and LOS were highest for heart disease (*n *= 8,450; LOS 6.2 days), pneumonia (*n *= 6,670; LOS 7.6 days), and malignant neoplasms (*n *= 2,225; LOS 9.8 days). The expenditure could be markedly reduced by even small changes in the readmission rate. Scrutiny of the factors associated with these readmissions may lead to identification of unmet clinical, educational, and psychosocial needs. An audit of 811 readmissions concluded that hospital system factors accounted for 37%, followed by clinical factors at 38%, and patient factors at 21% [32]. Once the causes of readmission are defined, research can focus on possible ways to improve the quality of care to reduce readmission rates, and consequently reduce the costs of readmission allowing relocation of resources.

### Strengths and Weakness of the Study

The main strength of this research lies in its large population from the CMS database of all public hospitals in Hong Kong provided by the HA which is responsible for 90% of hospital services in Hong Kong. The data are cross checked for completeness and accuracy.

Several limitations of this review should be considered. First, disease codes based on the primary discharge diagnosis were used, and the severity of disease and other co-morbidities were not available. Also, there may be a potential systematic difference or error in data entry of codes, as error rates are higher with more specific diagnostic codes [51]. However, it was believed that errors would be minimal in this study because all of the diagnostic codes were entered by the discharge physician. Second, while we explored the health outcome for various medical conditions in relation to readmission, data on the patients' quality of life, health status, functional status and satisfaction were unavailable. Finally, the cost data were incomplete because we were unable to access costs for follow-up, outreach community services, and human and other resources across various medical conditions; the overall costs may be higher than calculated. Nevertheless, the hospital cost, which contributed to the major portion of the total cost, was included.

## Conclusions

Our findings show that patient characteristics and geographical hospital cluster are correlated with 30-day unplanned readmission. After adjusting for these risk factors, patients with cancer, heart disease, pneumonia, liver diseases, and septicaemia were more likely to be readmitted. Readmission rates were associated with a longer hospital stay, higher mortality rate and greater hospital cost. A reduction in the risk of readmission would improve patient health and quality of life, with potential cost savings. To improve the quality of care, variations in individual diseases should be investigated with regard to the care process in hospitals and comprehensive discharge planning. Our findings indicate that a review is required of the care process for patients with malignant neoplasms, chronic liver disease and cirrhosis, septicaemia, heart disease and pneumonia, which have high readmission rates.

## Competing interests

The authors declare that they have no competing interests.

## Authors' contributions

ELYW supervised the data analysis and wrote the manuscript. AWLC analysed the data and MCML extracted the data. EKY supervised the data reporting. All authors contributed to the writing of the final draft of the manuscript. In addition, all authors read and approved the final manuscript.

## Pre-publication history

The pre-publication history for this paper can be accessed here:

http://www.biomedcentral.com/1472-6963/11/149/prepub
